# Spontaneous Transvaginal Small Bowel Evisceration With Uterine Procidentia: A Report of a Rare Case

**DOI:** 10.7759/cureus.27319

**Published:** 2022-07-26

**Authors:** Christopher J Pinto, Siddhi Hegde, Saraswathi Karekal, Djhemson Anneaud, Anagha Sharma

**Affiliations:** 1 Medical Research, Karnataka Insititute of Medical Sciences, Huballi, IND; 2 General Surgery, KVG Medical College & Hospital, Sullia, IND; 3 Obstetrics and Gynaecology, Karnataka Institute of Medical Sciences, Huballi, IND; 4 Anesthesiology, Washington University of Health and Sciences, Columbus, USA; 5 Obstetrics and Gynecology, Hassan Institute of Medical Sciences, Hassan, IND

**Keywords:** spontaneous presentation, post menopausal estrogen deficient, surgical gastroenterology, operative obstetrics, emergency obstetrics, rare case report

## Abstract

Spontaneous transvaginal small bowel evisceration is an extremely rare condition with a few more than 100 documented cases to date. The complications of such a rare entity revolve around its preoperative presentation as well as its operative and postoperative complications. The complications seen are intestinal incarcerations and perforations with an increased risk of post-surgical ileus and peritonitis in a time-dependent fashion. In our case, a postmenopausal female presented with sudden onset bowel evisceration through a defect in the posterior vaginal apex during straining for defecation. Past medical history was significant for intermittent abdominal pain, bloating, and chronic constipation. The patient had no signs of trauma, signs of sexual assault, preceding events, or prior urinary disturbances. The patient was treated surgically with laparotomy and bowel-packing followed by Ward-Mayo's repair with added on anterior and posterior colporrhaphy, and site-specific repair. Postoperatively, following a period of prolonged ileus, the patient made a full recovery. This case report aims to provide a better understanding of the mechanism and occurrence of such an event and also intends to raise awareness of this rare presentation as an emergency condition requiring prompt surgical management.

## Introduction

Spontaneous transvaginal small bowel evisceration is an extremely rare condition, which was first described in 1907 in a postmenopausal female [1]. Though evisceration of the abdominal contents has been noted through defects in the urogenital vault through the rectum, uterus, and vaginal canal, there are only a few more than 100 documented transvaginal cases to date [2]. Many reports present as an outcome of obstetric procedures leading to iatrogenic presentations; however, there exists documentation of a smaller proportion of such, occurring without the preceding history of instrumentation [1-3]. As seen in this case, a postmenopausal state with decreased estrogen could cause atrophy of the urogenital diaphragm, laxity of the pelvic support structures, and ulceration of the vaginal mucosa causing a lead point for the evisceration [4,5]. Uncommon precipitating factors could include conditions that increase intra-abdominal pressure: chronic cough, constipation, and weight lifting [3]. Following prompt surgical correction, the mortality is estimated to be at 6-10% attributed to septicemia and thromboembolism following the restoration of the blood supply to the ischemic bowel, hence, requiring a multi-disciplinary approach in primary prevention and postoperative recurrence [6].

## Case presentation

History and physical examination

An Indian female, aged 63 years, was brought to our Emergency Department at Hubballi, Karnataka, by her family members, after they found her on the washroom floor, a few minutes after she went in to relieve herself. The patient mentioned that while she went to defecate, while straining, she felt her abdomen give way, after which she lost consciousness for a brief moment. Obstetric and Gynecological history was para 2, living 2 (P2L2), both full term vaginal deliveries in the patient's early 20s. Though medical records were not available, the patient recalled that she had postpartum hemorrhage due to multiple vaginal tears during her second delivery. The patient was not sexually active for the last eight years. The patient reports that she did not endure any injuries to her perineum in the recent past. Past medical history was significant for intermittent constipation, bloating, and abdominal pain for the last two years, which was diagnosed as constipation-predominant irritable bowel syndrome (IBS) at a private clinic. The patient was asked by her primary care physician to make dietary modifications to counter her IBS, which did not help in alleviate her symptoms. The patient was then prescribed bisacodyl suppositories with moderate improvement of her symptoms.

**Figure 1:**
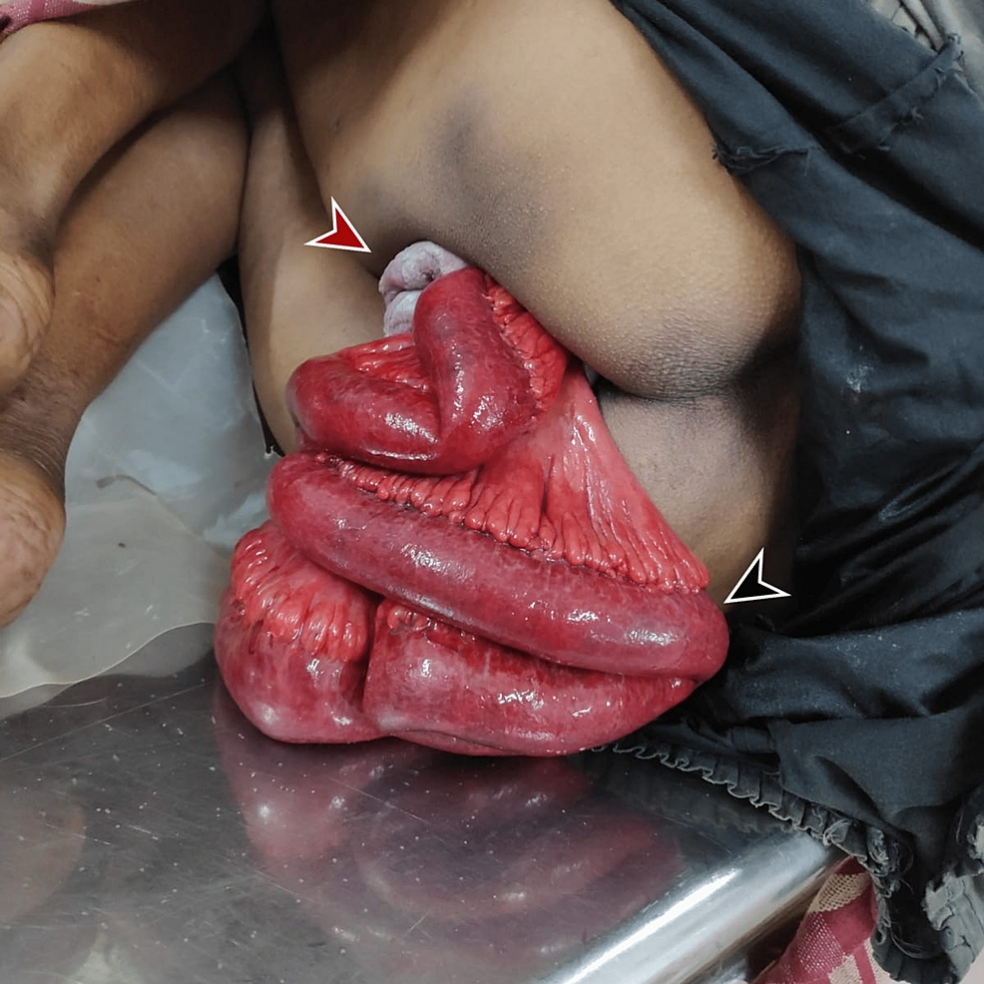
Physical findings at presentation. The multiparous cervix with the uterus (marked in red) with erythematous pink bowel projection transvaginally (marked in black).

The patient's vitals were 106/64 mmHg with a heart rate of 124 bpm, a respiratory rate of 40 cycles per minute, and a saturation of 90%. At presentation, the patient was anxious and restless. Physical examination showed visible small bowel of length of 80 cm eviscerating trans-vaginally. The bowel was pink, peristaltic and protruding from the vaginal orifice with no bleeding or visible perforations. Anterior to the small bowel, a multiparous cervix with grade 4 uterine prolapse was noted as seen in Figure 1. There were no signs of trauma or sexual assault on the patient. Palpation of the abdomen and hernial orifices were contraindicated lest incarceration or prolapse of the bowel occurred.

Treatment

ED Course

The patients' bowels were washed with normal saline and were then wrapped with warm saline soaked gauzes under aseptic conditions. The patient was started on intravenous midazolam to counter the presumed anxiety-induced tachypnea, which did not reduce the respiratory rate. Following this, the patient was given 10L O_2_ through a simple face mask. Respiratory distress due to sudden depressurization of the abdominal cavity was suspected [7,8]. Obstructed bladder outlet together with the inability to catheterize due the procidentia, contraindicated the use of high-volume fluid therapy. The patient was started on piperacillin, tazobactam, and metronidazole preoperatively. Necessary consent for bowel surgery, hysterectomy, and site-specific repair was taken as a part of emergency-surgical intervention.

Surgical Course

**Figure 2:**
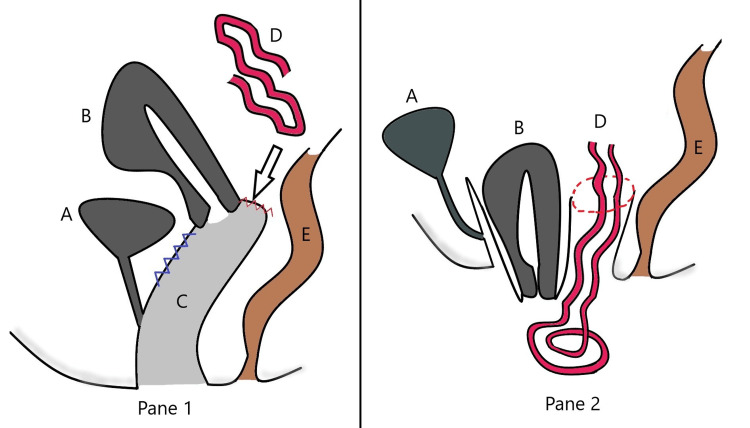
Pictorial representation of the defects noted. Pane 1: The site of anatomical defect at the posterior vaginal apex (marked in red zig-zag line) with laxity of the anterior vaginal wall (marked in blue zig-zag). Pane 2: Current presentation of the patient A: Bladder, B: Uterus, C: Vagina, D: Small bowel, E: Rectum Image credit: Dr. Christopher Jude Pinto

A General Surgeon team was invited to partake in the surgical management of the patient and provide assistance during the course of the surgery. The bowel was peristaltic on preoperative repeat saline wash. Under general anesthesia, through a midline abdominal incision, the bowels were gently retracted into the abdominal cavity. The entire length of the bowel was examined and showed no perforations, leaks, tears or areas of devitalization. Following bowel inspection and closure of the abdominal wall, the patient was placed in the dorsal lithotomy position for the obstetric correction. Following manual reduction of the prolapsed uterus, a large defect measuring 7*6cm was noted at the posterior fornix of the vagina. A pictorial representation of the site of the defect was noted as in Figure 2.

**Figure 3:**
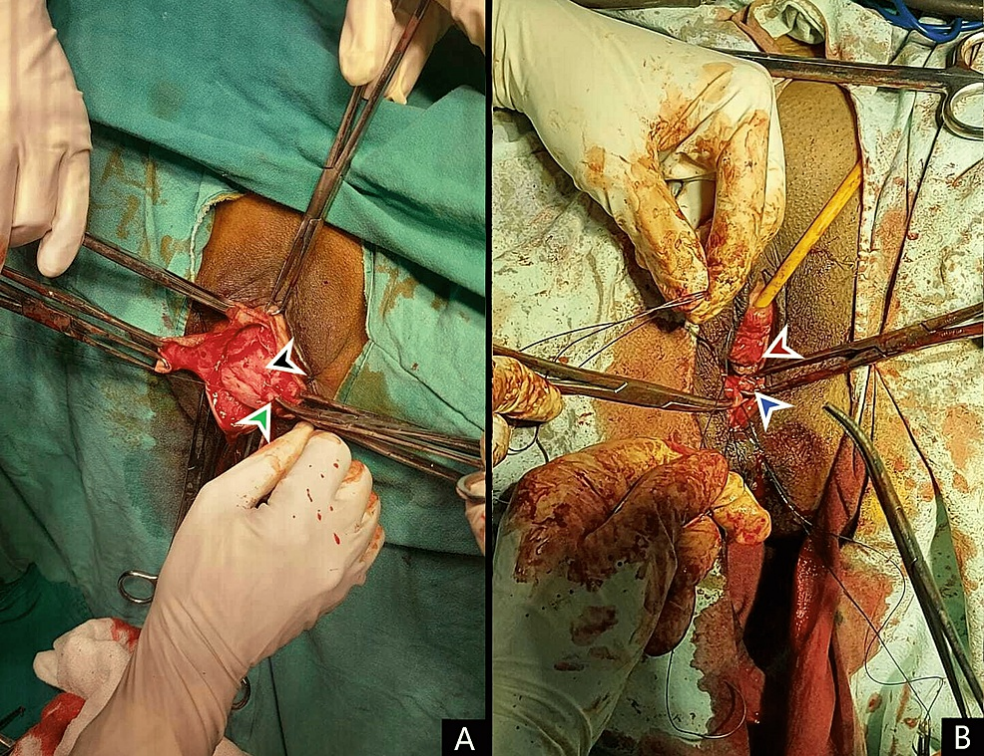
A. Anterior colporrhaphy: step of bladder delineation (marked in black) and plication suture application (marked in green). B. Post hysterectomy fixation of the apex of the posterior vaginal wall (marked in red) with the resected and newly plicated posterior wall (marked in blue)

A decision to repair the grade 4 uterine uterine prolapse with Ward-Mayo's repair vaginal hysterectomy with combined anterior and posterior colporrhaphy was made and performed (Figure 3). Site-specific repair of the defect was done by removal of the excess lax tissue and plication of the muscularis layers. Following the site-specific repair, an added procedure of sacrospinous fixation was done with prolene 2-0.

Postoperative course and follow-up

The patient was reserved for another emergency laparotomy lest signs of peritonitis were observed. Post the surgical correction, the respiratory rate and saturation showed marked improvement and normalization. The patient's postoperative period was complicated, with prolonged ileus for five days. In the interim, the patient was supplemented parenterally with IV fluids. Medication management with metoclopramide helped relieve the ileus, after which the patient passed flatus and stools. The patients antibiotics were continued till day seven of inpatient care and was discharged on day 11. Monthly follow-ups thereafter showed well healing margins without any further recurrence. Patient also reported resolution of the prior bloating and the constipation, without the use of suppositories.

## Discussion

Spontaneous transvaginal bowel evisceration is a rare emergency surgical condition, with the at-risk groups including the elderly (postmenopausal) and premenopausal females with a prior positive surgical history [5,9]. In the premenopausal population, following hysteroscopic or vault surgeries, defects in fascia repair could be a lead point for evisceration of abdominal contents [4,10]. This mechanism of evisceration was first described in the 19th century through a rent in the vaginal wall leading to the prolapse of the abdominal contents [1,3]. In 1907, another report described spontaneous evisceration in a 63-year-old while weight lifting [1,3]. Vaginal trauma in the form of insertion of foreign bodies and direct trauma could also be a cause for the evisceration, indicating the need for sexual assault history [4]. A history of enterocoele, ulcerative gynecological neoplasms/lesions, and multiparity may also be seen in such patients [6,11,12]. In the majority of all eviscerations, 50-75% of cases have shown a prior history of vaginal surgery with 25% related to postoperative hysterectomy sequelae [6,11-13].

In a postmenopausal female, reduced estrogen could cause atrophy of the urogenital diaphragm and pelvic support structures due to diminished vascularity, making the structures lax and prone to prolapsing through the vaginal wall [2,5]. Compounding factors such as weight lifting, falling, chronic coughing, and chronic constipation could further lead to enlargement of the rent with further progression leading to an active presentation [2,4,5,14]. The mechanism of injury can be explained by the levels of vaginal support as specified by Delancey. Level 1 is located at the apex of the vagina with the support provided by the uterosacral-cardinal complex. Level 2 support is through the arcus tendinous fascia pelvis (anterior vagina) and arcus tendineus of the levator ani (posterior vagina) providing support to the middle third of the vagina. Level 3 support is through the urogenital diaphragm and perineal body to the distal part of the vagina. In the current case, the presentation could have been an outcome of having all the levels of support lost compounded by the increased intra-abdominal pressure due to chronic constipation and straining for defecation [15]. An unrepaired defect during parturition could have created a weak scar. This scar, in a postmenopausal state, could have also led to a quiescent enterocoele leading to intermittent symptoms of small bowel obstruction as noted in our case. Moreover, if the risk factors co-exist together, (i.e., the postmenopausal state with constipation and a quiescent enterocoele) this outcome, with both uterine prolapse and bowel evisceration, becomes the more likely the explanation for the current presentation. The role of obstetric history is vital is determining the possible etiology and, in the current case, the history of postpartum hemorrhage due to multiple vaginal tears helped provide a possible etiology.

A variety of approaches have been demonstrated in literature: abdominal, vaginal, and laparoscopic, combined abdominal-vaginal, or combined laparoscopic-vaginal. The selection of approach depends on the viability of the prolapsed bowel and mesentery, accessibility through the vaginal route, and the surgeon's ability to replace the prolapsed bowel [2,4-6,16]. Robotic surgery has also shown successful management with better visualization and easier maneuvering of the organs [17]. Following hemodynamic stabilization of the patient, an attempt may be made to reposition the bowel intraperitoneally but if it fails, it should be immediately followed by surgical repair [18]. The principle of management revolves around the viability of the bowel, if incarcerated, the bowel has to be resected but if it's viable, resection is not necessary in early presentations [19,20]. In the present case, pink bowels, with peristalsis indicated viability and, hence, indicated only gynecological repair. To avoid future vault prolapses in cases that are high risk identified during a hysterectomy with noticed laxity in the layers of support, preventive sacrospinous fixation and sacrocolpopexy can be effective [18]. In this case, we chose to perform Ward-Mayo's repair with sacrospinous fixation by using a prolene pulley and anterior and posterior colporrhaphy with Vicryl (Ethicon Inc., Raritan, New Jersey, United States) to repair and reinforce the posterior vaginal apex.

Medical management is also crucial in the overall recovery of the patient. The current patient had respiratory distress indicated by a high respiratory rate as a result of the evisceration due to the loss of abdominal pressure leading to the flattening of the diaphragm [7,8]. Respiratory management with intranasal oxygen, anxiolytics, pain medication (non-opiates, as opiates are respiratory depressants), and general anesthesia with intubation are all steps to manage the patient preoperatively. Postoperatively, management should revolve around the patient primarily passing stools, as in our case, simple surgical repositioning helped resolve the constipation by elimination of the quiescent enterocoele. Management of the postoperative ileus by prokinetic agents helped in the course of resolution and acted as an indication of the viability of the bowels. Due to the associated rarity and complications by the delay in proper treatment, it is imperative to use a multidisciplinary approach involving obstetricians, general surgeons, and an internist team, for patients with bowel evisceration to make a complete and successful recovery [6,14].

## Conclusions

Spontaneous transvaginal small bowel evisceration is an extremely rare presentation often seen in postmenopausal females or as an outcome of obstetric surgery in the premenopausal age group. Early ED presentations of the condition could show pink peristaltic bowels in conjunction with other organ prolapses. The complications could include intestinal incarceration and intestinal perforation with an increased risk of post-surgical peritonitis in a time-dependent fashion. Choice of surgical intervention procedure is decided upon the degree of incarceration and time of presentation from the inciting event. A multidisciplinary approach involving general surgeons, obstetricians, and an internist team is required for the successful recovery of a patient, with regular follow-ups to check for recurrence.
